# Remdesivir Use in Pediatric Patients with Acute SARS-CoV-2 Infection Is Safe and Well Tolerated

**DOI:** 10.3390/children12030331

**Published:** 2025-03-06

**Authors:** Delma J. Nieves, M. Tuan Tran, Jasjit Singh, Negar Ashouri, Tricia Morphew, Jennifer G. Lusk, Felice C. Adler-Shohet, Rachel Marano, Stephanie Osborne, Jennifer Strickland, Antonio C. Arrieta

**Affiliations:** 1 Division of Infectious Diseases, Department of Pediatrics, CHOC Children’s Hospital, University of California, Orange, CA 92868, USA; jsingh@choc.org (J.S.); nashouri@choc.org (N.A.); aarrieta@choc.org (A.C.A.); 2 Division of Infectious Diseases, Department of Pharmacy, CHOC Children’s Hospital, Orange, CA 92868, USA; mtran@choc.org; 3 CHOC Research Institute, Orange, CA 92868, USA; tricia@morphewconsulting.com (T.M.); sosborne@choc.org (S.O.); 4 Division of Hospitalist Medicine, Department of Pediatrics, CHOC Children’s Hospital, University of California, Orange, CA 92868, USA; jlusk@choc.org; 5 Harbor-UCLA Medical Center, Torrance, CA 90509, USA; felice.adler@lundquist.org; 6 Rady Children’s Hospital, University of California, San Diego, CA 92123, USA; rmarano1@rchsd.org; 7 Division of Infectious Diseases, CHOC Children’s Hospital, Orange, CA 92868, USA; jennifer.chrislip@choc.org

**Keywords:** Remdesivir, pediatric COVID-19, SARS-CoV-2, antiviral

## Abstract

**Background/Objective:** Millions of children were infected with SARS-CoV-2, and a small proportion progressed to severe disease, especially those with underlying risk factors. Adult COVID-19 studies showed mortality benefits with Remdesivir. Data on Remdesivir use in pediatrics are limited. We report on the safety and tolerability of Remdesivir in pediatric patients seen at our institution. **Methods:** This was a retrospective cohort study of patients <19 years old with acute SARS-CoV-2 infection who received at least one dose of Remdesivir. Patients followed strict institutional guidelines for safety monitoring including standard clinical and laboratory daily observations. Demographics and underlying conditions were reported as averages; for laboratory values, linear regression was applied within a generalized linear mixed-effects model framework to evaluate the significance of changes in average levels over time. **Results:** We enrolled 318 patients with acute SARS-CoV2 infection from May 2020 to December 2022. In total, 53% were male, and the age range was distributed broadly. In total, 61% were school-aged children (28% 5–11 and 33% 12–18 years of age). In total, 62% of cases were Hispanic. The most common reasons for Remdesivir treatment included respiratory distress (201; 63%) and having high-risk underlying conditions (109; 34%). Therapy was completed as planned in 91% and discontinued early in 9%. Mean baseline, peak, and end of treatment values for AST were 57 (95% CI 53, 61), 79 (95% CI 73, 84) (*p* < 0.001), and 55 (51, 59) (*p* = 0.479); for ALT, they were 42 (38, 47), 59 (95% CI 52, 66) (*p* < 0.001), and 46 (95% CI 41, 52) (*p* = 0.054); and for bilirubin, they were 0.56 (95% CI 0.50, 0.62), 0.67 (95% CI 0.61, 0.74) (*p* < 0.001), and 0.44 (95% CI 0.40, 0.48) (*p* < 0.001), respectively. During Remdesivir treatment, we did not observe marrow suppression or renal toxicity. **Conclusions:** No clinically significant hematological or renal toxicity was noted. Mean liver enzymes increased modestly and returned to baseline without interrupting treatment. Remdesivir was well tolerated in patients <19 years old.

## 1. Introduction

Since the onset of the COVID-19 pandemic, >15 million cases have been reported in children in the United States representing close to 20% of all cases, with 5% requiring hospitalization mainly those with underlying high-risk conditions [1,2,3,4,5]. Remdesivir in its active form (Remdesivir triphosphate) is a SARS-CoV-2 ribonucleic acid polymerase inhibitor [6]. Adult studies showed mortality benefit in those requiring oxygen, but elevation of liver enzymes was observed [7,8]. Other studies suggested decreased glomerular filtration rate/increased creatinine, a decrease in hemoglobin and absolute lymphocyte count and even bradycardia of no clinical significance observed in previous pediatric trials [9,10,11,12]. Based on randomized adult clinical trials, which showed improved survival, particularly in patients receiving oxygen who were treated for 5–10 days depending on the severity of illness or underlying medical conditions, in October 2020, the U.S. Food and Drug Administration (FDA) approved the use of Remdesivir for patients >12 years old and >40 kg requiring hospitalization for acute SARS-CO-V2 infection and gave emergency use authorization for patients <12 years and >3.5 kg. Following a large randomized controlled trial evaluating the efficacy of Remdesivir outpatient treatment for 3 days for preventing hospitalization in adult patients at high risk for progression to serious illness, on 21 January 2022, it was approved for use in patients >12 years old with mild-to-moderate COVID-19 at high risk for disease progression (3-day regimen) [13]. On 25 April 2022, it was approved for use in adults and pediatric patients aged >28 days and weighing >3 kg. Few reports of pediatric use of Remdesivir exist and these are mainly small, single-center, retrospective studies.

Here, we present the largest to date cohort of pediatric patients receiving Remdesivir for treatment COVID-19 of different degrees of severity, treated for 5–10 days, and patients without evidence of lower respiratory tract disease but at risk for progression to severe disease who receiving an abbreviated (planned 3 days) course of Remdesivir. The aim of this study was to evaluate the safety and tolerability of Remdesivir in children and adolescents.

## 2. Materials and Methods

This is a single-center retrospective cohort study from Children’s Hospital of Orange County, California. All patients from birth to <19 years of age with confirmed SARS-CoV-2 infection who received at least 1 dose of Remdesivir were eligible. Acute SARS-CoV-2 infection was confirmed by PCR using BioFire RP2.1, a real-time, nested multiplexed polymerase chain reaction test or Cepheid Rapid PCR detection tests. All patients were followed by the infectious diseases team; they were treated and monitored following an institutional guideline: (A) Remdesivir was given as a loading dose of 5 mg/kg up to 200 mg followed by maintenance of 2.5 mg/kg up to 100 mg daily. (B) All patients received at baseline, and daily while on treatment, a set of laboratory tests, which included (i) complete blood cell count (CBC), including absolute neutrophil count (ANC), absolute lymphocyte count (ALC) and platelet count, (ii) complete metabolic panel, which included electrolytes, creatinine, aspartate aminotransferase (AST), alanine aminotransferase (ALT) and bilirubin, (iii) coagulation studies including prothrombin time (PT) and D-Dimers. Baseline (before initiation of Remdesivir), peak or nadir (maximum or minimum value during treatment) and end-of-therapy (EOT) values were used to evaluate Remdesivir safety. (C) Patients who required oxygen support were on continuous oxygen saturation monitoring, which provides continuous heart rate monitoring, otherwise they had their vital signs monitored every 4 h. (D) Patients had to have AST, ALT, and bilirubin <5 times upper limit of normal (ULN) to initiate Remdesivir treatment. If liver enzymes increased to >10 ULN while on Remdesivir, treatment was discontinued. (E) Early in the pandemic, patients who had severe disease (required ventilatory support; invasive or non-invasive) or who had serious underlying conditions (transplant recipients, oncology patients, primary immune deficiencies, chronic lung or cardiac disease requiring ongoing oxygen therapy) received 10 days of treatment; all others (healthy patients requiring oxygen at baseline, patients underlying risk factors not meeting above criteria such as morbid obesity, neuromuscular disorders, diabetes, chronic lung disease, cardiac disease, asthma, chronic kidney disease) received a 5-day regimen.

Starting in January 2022, following the publication by Gottlieb et al. using early Remdesivir for prevention of progression of disease in patients with at least one risk factor for disease progression [13], patients with SARS-CoV-2 infection and any of the underlying risk factors described above or healthy patients with lower respiratory tract signs and symptoms, not meeting COVID-19 criteria (croup, bronchiolitis) received a 3 days course of Remdesivir unless they failed to improve, in which case they would be reassigned to a 5-day regimen.

Baseline demographic data, oxygen requirement, method of delivery, and medical history with special emphasis on previously described conditions associated with severe illness were obtained.

All patients with COVID-19 received vitamin D and enoxaparin; glycemia was monitored every 4 h and if it reached >160 mg/dL for 2 measurements, and an insulin drip was started.

Statistical analysis was completed as follows. Demographic characteristics and underlying diagnoses of the patient population were summarized using counts and percentages for each defined trait. The percentage of patients requiring respiratory support prior to Remdesivir treatment, at the end of treatment, and at discharge was compared across repeated measurements using a generalized linear mixed-effects model (GLMM) with a specific binomial distribution, an identity link function, and an unstructured covariance matrix. Laboratory values were evaluated to determine appropriate distributional assumptions for comparing average values prior to treatment with peak (or nadir) levels observed during treatment and by the end of treatment. Extreme values were excluded before assessing average values across time; no more than two patients were excluded for any specific laboratory result at any time point. For most laboratory values, a normal distribution was assumed, and linear regression was applied within the GLMM framework to evaluate the significance of changes in average levels over time. However, for ALC, creatinine, and ALT, which exhibited significant skewness, gamma regression was utilized. Estimated mean laboratory values at each time point were generated from the GLMM models with corresponding 95% confidence intervals. The significance of pairwise comparisons applied the least significant difference adjustment for multiple comparisons. Adjusted analyses were also performed to account for potential confounding factors, including sex (male/female), ethnicity (Hispanic/Non-Hispanic), age group (<12 years, ≥12 years), presence of an underlying condition, and days of planned treatment (≤3 vs. >3 days). All analyses were performed using SPSS version 29.0.

Although this is not a comparative trial and we did not have a control arm, outcome data were captured including discharge alive, discharge on oxygen (if not on oxygen prior to infection), pediatric intensive care unit admission, invasive ventilatory support, length of in-hospital stay, unplanned discontinuation of treatment and reason for discontinuation (death, adverse events, improvement, other).

## 3. Results

All 318 patients hospitalized with SARS-CoV-2 infection, who received Remdesivir from May 2020 through December 2022, were included in the present study. Fifty-three percent of patients were male. The age range was distributed broadly with 61% being school-aged children (28% 5–11 and 33% 12–18 years of age). Hispanics accounted for 62% of cases. The median length of the in-hospital stay was 5.0 days (IQR: 4.0, 9.0; min 1, max 123). Most cases (87%) had an underlying diagnosis considered to put them at increased risk for COVID-19 complicated disease. Details are presented in Table 1. The indication for treatment was listed as respiratory distress in 201 (63%), considered high risk in 109 (34%) and other in 8 (3%). The eight patients with “other” were one with encephalopathy, five due to fever, one pre-operative and one multisystemic inflammatory syndrome.

Remdesivir was completed as planned, based on an indication at the start of treatment (see below) in 290 (91%) patients. Since some patients had Remdesivir discontinued before completing the planned length of treatment, the duration of treatment for the whole cohort was between 1 and 10 days. When used for patients hospitalized due to respiratory distress with acute COVID-19, Remdesivir was planned for 5 doses and extended for up to 10 in severe cases. When prescribed as early treatment for prevention of progression of disease in high-risk patients, Remdesivir was planned for three doses. The majority (87%) of patients received three doses (154 patients) or five doses (124 patients). Ten patients received 10 doses, three received 6 to 8 doses, sixteen received 4 doses, three received 2 doses and eight received 1 dose. Remdesivir was discontinued earlier than planned in 28 (9%) patients. The reasons for early discontinuation were improved/ready for discharge (15), experienced liver enzyme elevation (6), bradycardia (1), parent request (1), IV infiltrated (1), peritoneal dialysis (1), transferred out (1), did not return for dose (1) and death (1). Of the patients for whom Remdesivir was discontinued due to elevated liver enzymes, neither of them met Hy’s Law criteria for drug-related hepatotoxicity. One was a 7-week-old infant with multiple congenital abnormalities; at the time Remdesivir was discontinued, his total bilirubin was 1.9 mg/dL (alkaline phosphatase was normal). The second one was a 3-year-old girl with Dravet’s syndrome, on multiple anticonvulsant agents (phenobarbital, clobazam and diazepam as needed), who developed elevated liver enzymes > 10 times ULN; after the first dose of Remdesivir, she had normal bilirubin (0.3 mg/dL) and elevated alkaline phosphatase at the time.

**Figure 1:**
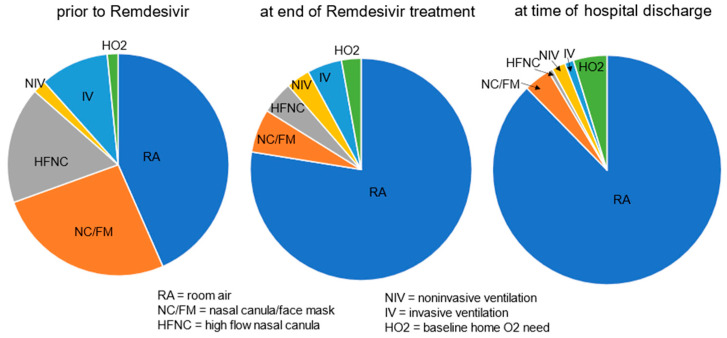
Respiratory support needs [RA/none vs. any (end of Tx and discharge compared to prior to start of treatment, *p* < 0.001)].

More than half of cases (180 (57%)) had a new or increased need for respiratory support at the start of Remdesivir treatment, including 83 (26%) on nasal canula O_2_, 54 (17%) on high flow nasal canula, 32 (10%) with invasive and 6 (2%) with noninvasive ventilation support, respectively, and 5 (1.6%) were on home O_2_ at baseline. At the end of Remdesivir treatment, 23% of cases continued to have an increased respiratory support need from baseline but by the time of discharge, it was down to 12% (Figure 1). The decreased need for respiratory support, from 57% prior to the start of treatment to 23% by the end of treatment, and further to 12% by discharge was significant (*p* < 0.001).

Several clinical laboratory tests were followed during Remdesivir treatment. These include baseline (before Remdesivir was started), during treatment and at EOT measurements for WBC, ANC, ALC, platelets, creatinine, AST, ALT, total bilirubin, and prothrombin time (INR). For clinically relevant tests, the peak or nadir values were also collected (Table 2).

**Figure 2:**
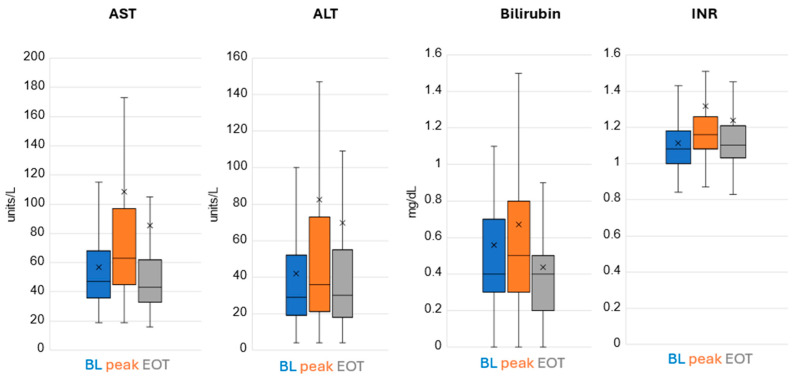
Hepatic Labs. Lower and upper box boundary 25th and 75th percentiles, respectively; inside line box median, lower and upper error line 10th and 90th percentiles, respectively. X is the mean.

Remdesivir treatment did not result in marrow suppression nor renal toxicity. There was a mild degree of hepatic enzyme elevation noted in a few patients with the vast majority tolerating treatment very well (Figure 2).

The AST value was 5–10 times the upper limit of normal in 0.9% of patients at baseline, increased to 1.9% of patients during treatment and was back to 0.6% by EOT. The AST value was >10 times the upper limit of normal in 0% of patients at baseline, increased to 0.6% of patients during treatment and stayed at 0.6% by EOT (treatment was stopped in these patients due to elevated liver enzymes >10 times ULN; hence, this is the EOT value). The ALT value was 5–10 times the upper limit of normal in 1.3% of patients at baseline, increased to 4.8% of patients during treatment and was at 3.9% by EOT. The ALT value was >10 times the upper limit of normal in 0.3% of patients at baseline, increased to 1.3% of patients during treatment and decreased to 0.6% by EOT.

There were 148 patients who completed a planned 3 days of Remdesivir for early treatment to reduce the risk of developing severe disease. Their underlying risk factor(s) were listed as none in 16, neuromuscular in 18, immunodeficiency in 11, oncologic in 26, obesity in 3, chronic lung disease in 11, a combination of these in 37 and other in 26. At the time of presentation, 70% were on room air or baseline home ventilator settings with that increasing to 96% by the time of discharge. Hepatic laboratory values showed a significant increase at their peak levels compared to pre-treatment (*p* < 0.05) but returned to baseline levels by end of treatment (*p* > 0.05), except for bilirubin, which was reduced to below the average baseline level (mean baseline, peak and EOT values for AST were 57 (95% CI 53, 61), 79 (95% CI 73, 84) (*p* < 0.001), 55 (51, 59) (*p* = 0.479); for ALT 42 (38, 47), 59 (95% CI 52, 66) (*p* < 0.001), 46 (95% CI 41, 52) (*p* = 0.054); and bilirubin 0.56 (95% CI 0.50, 0.62), 0.67 (95% CI 0.61, 0.74) (*p* < 0.001), 0.44 (95% CI 0.40, 0.48) (*p* < 0.001), respectively). Adjusting for potential confounding factors did not change the direction or significance of the findings, Table 3.

A sub-population analysis was performed after excluding patients whose AST and ALT were at their peak levels at baseline, as shown in Table 4.

In this sub-population, average AST and ALT levels were significantly elevated at their peak compared to baseline [mean difference (peak-baseline): AST 35.2 (95% CI 29.1, 41.3), *p* < 0.001; ALT 27.8 (95% CI 20.3, 35.3), *p* < 0.001]. By the end of treatment, both AST and ALT levels showed a significant downward trend from their peak values [mean difference (peak-EOT): AST 21.3 (95% CI 15.8, 26.7), *p* < 0.001; ALT 9.8 (95% CI 4.4, 15.3), *p* < 0.001)].

Two patients died during hospitalization. Both had severe underlying abnormalities. Remdesivir was not thought to contribute to either death.

## 4. Discussion

Here, we present the largest-to-date report on the safety and tolerability of Remdesivir, an antiviral agent with demonstrated clinical activity against the SARS-CoV-2 virus in pediatric patients. This agent was demonstrated to reduce mortality in adults with moderate to severe respiratory disease and to prevent progression to serious respiratory illness due to SARS-CoV-2 in patients at risk for COVID-19 [7,13]. Our findings support previous smaller retrospective reports in pediatric patients [14,15,16].

The COVID-19 pandemic led to rapid adaptation and adoption of novel therapies. Pediatric patients were broadly affected with some more susceptible to complications than others. Children with obesity, prematurity, chronic lung and cardiac disease, diabetes and immunosuppression were noted to have more complicated or prolonged disease. Early in the pandemic, Remdesivir was found to improve survival in COVID-19 adult patients with moderate to severe disease [17]. Some safety signals were noted in early clinical trials, particularly hepatobiliary and renal toxicities were reported. Considering the seriousness of COVID-19 in adults, these toxicities were considered acceptable, but close monitoring was suggested [7]. With extrapolation from adult data, it was rapidly approved for use in pediatrics initially as an emergency Investigational New Drug and later officially given FDA approval. Considering the milder disease and lower mortality rate in pediatric patients, it became imperative to conduct studies evaluating the safety of this agent in this fragile population.

To date, no randomized controlled trials on the safety and efficacy of Remdesivir in children have been reported. In 2022, Samuel et al. reported their experience with Remdesivir on a small single-center retrospective trial that included 48 patients [14]. Even though this was not a comparative trial, they used their patient’s clinical course to provide some perspective on the efficacy and safety of Remdesivir treatment; importantly, they reported bradycardia in 20% of patients with half of them experiencing hypertension, none of these had to discontinue therapy. None of their patients experienced hepatic or renal toxicity. In a separate single-center retrospective study in Poland, Kautsch et al. reported on 259 pediatric patients with SARS-CoV-2 infection; 64 of them received Remdesivir, and only 4 of these experienced elevation of transaminases of <3 times ULN, none of which required treatment discontinuation. They did not observe bradycardia or signs of AKI [18]. Creatinine elevation in 16% of 176 patients on whom creatinine was measured during treatment was reported by Player et al. in a larger single-center retrospective study that enrolled 180 patients. The authors also reported elevated transaminases in 60 (34%) patients who had liver enzymes measured during treatment; 14 (7.7%) had grade 3–4 elevation, 12 of whom had transaminase elevation at baseline. The authors do not report if treatment had to be stopped in any patients [19]. Romani et al., in a multicenter retrospective study, reported their experience in 50 patients identified in 10 centers in Italy; there was no difference in median transaminase values at baseline or during treatment, and they found no increase in creatinine. Bradycardia was reported in 16 patients [20]. Similar to these reports, ours is a retrospective study, but unlike previous reports, our patients were closely followed clinically and, per institutional guidelines, received daily safety laboratory monitoring, which allows us to have an accurate perspective of the day-to-day impact of Remdesivir exposure. Our findings, in a larger population, are similar to those presented in these studies; we found mild, not clinically significant elevation of transaminases and bilirubin during treatment which returned to baseline by EOT; only two patients experienced serious hepatotoxicity probably related to Remdesivir, both had significant pre-existing comorbidities, including a 7-week-old patient with multiple congenital anomalies and a 3-year-old with metabolic disorder. Remdesivir was stopped and liver enzymes and bilirubin returned to normal; only one patient in our cohort experienced bradycardia. No renal toxicity or coagulopathy were noted. We did not see evidence of bone marrow toxicity.

We are the first to present data on the safety of Remdesivir utilization for prevention of progression of disease in pediatric patients with serious underlying conditions and for treatment of less severe syndromes reported with newer SARS-CoV-2 variants such as bronchiolitis and croup.

Remdesivir treatment was completed as planned, based on indication for treatment, in 91% of patients. The treatment duration was most often 5 days for children with acute disease demonstrating respiratory distress or 3 days for those with early identified infection and considered high risk for disease progression. Treatment was discontinued earlier than planned in 28 (9%) patients, and only 7 (2.2%) patients had their treatment discontinued due to adverse events (liver enzyme elevation 6, bradycardia 1).

Most patients had significant clinical improvement during their hospitalization, although efficacy could not be assessed in this single-arm study. Most patients were discharged without an oxygen requirement. Mortality was low (0.63%) and not assessed as drug-related.

The most important limitation of our study is the absence of a control arm that would allow us not only the ability to assess Remdesivir efficacy but importantly ascertain if some of the adverse events described in our study are associated with SARS-CoV-2 infection or with the serious underlying diseases frequent in our cohort rather than to Remdesivir toxicity. Another important limitation is the retrospective nature of our study which limits our ability to discriminate for the presence of groups at higher risk for complications. The most important advantage of our study is the large cohort (318 patients), which is larger than previous pediatric trials. Another important advantage is that since we adopted Remdesivir treatment early in the pandemic, before much information was available, we developed a careful monitoring protocol to identify safety concerns rapidly as we used the agent, which has allowed us to report with significant granularity on the safety and tolerability of Remdesivir in pediatric patients, including those with severe comorbidities.

As the pandemic wanes and emerging variants appear to be less virulent one may wonder about the timeliness of our study. Scientific evidence suggests that beta-coronaviruses will emerge again in the future, potentially posing an existential threat [21]; therefore, it is imperative to increase our knowledge on treatments for clade 1-sarbecoviruses while pansarbecovirus vaccines are developed.

## 5. Conclusions

In conclusion, our data, which include a large number of patients of a broad age range and diverse racial and ethnic backgrounds, add to the existing knowledge of Remdesivir, an important antiviral agent in pediatric patients, confirming information presented recently by others on the acceptable safety and tolerability of this agent in healthy children and those with serious comorbidities. It suggests that not only is Remdesivir useful for the treatment of severe SARS-CoV-2 lower respiratory tract infection, but short courses of treatment, started early in the course of the disease, are safe and may be efficacious in preventing progression to serious illness as has been documented in adult studies.

## Figures and Tables

**Table 1:** Demographics.

	Cases
Age	
<12 months	41 (13%)
1–2 years	47 (15%)
3–4 years	35 (11%)
5–11 years	89 (28%)
12–<19 years	106 (33%)
Sex	
Female	148 (47%)
Male	170 (53%)
Ethnicity/Race	
Hispanic	197 (62%)
Non-Hispanic White	64 (20%)
Pacific Islander	2 (1%)
Asian	16 (5%)
Black	9 (3%)
Other	27 (8%)
unknown	3 (1%)
Underlying Diagnosis	
None	42 (13%)
Neuromuscular	92 (29%)
Immune deficiency	30 (9%)
Oncological	48 (15%)
Chronic lung disease/Asthma	86 (27%)
Obesity	36 (11%)
Other	89 (28%)
Multiple	89 (28%)

**Table 2:** Unadjusted multivariate model a: average values at baseline, min or max during treatment, and at the end of treatment ^a^.

	Baseline Mean (95% CI)	Peak ^P^ or Nadir ^N^ Mean (95% CI)	End of Treatment Mean (95% CI)	*p*-Value ^b^
WBC (K/UL) ^(N)^	8.0 (7.2, 8.8)	5.2 (4.9, 5.5) ^^N^	7.3 (6.8, 7.8) ^ns^	<0.001
ANC (count/UL) ^(N)^	5217 (4779, 5655)	2587 (2340, 2834) ^^N^	3943 (3549, 4337) ^^^	<0.001
ALC (count/UL) ^(N)^	1898 (1638, 2200)	1413 (1259, 1586) ^^N^	2736 (2495, 3000) ^^^	<0.001
Platelets (K/UL) ^(N)^	235 (221, 249)	204 (191, 217) ^^N^	271 (254, 288) ^^^	<0.001
Creatine (mg/dL) ^(P)^	0.39 (0.35, 0.42)	0.43 (0.39, 0.47) ^^P^	0.36 (0.33, 0.39) ^^^	<0.001
AST (units/L) ^(P)^	57.0 (52.7, 61.3)	78.5 (72.6, 84.3) ^^P^	55.1 (51.0, 59.2) ^ns^	<0.001
ALT (units/L) ^(P)^	42.0 (37.6, 46.9)	58.7 (52.2, 66.1) ^^P^	46.4 (41.3, 52.0) ^ns^	<0.001
Bilirubin ^(P)^	0.56 (0.50, 0.62)	0.67 (0.61, 0.74) ^^P^	0.44 (0.40, 0.48) ^^^	<0.001
INR ^(P)^	1.11 (1.09, 1.13)	1.30 (1.17, 1.43) ^^P^	1.22 (1.09, 1.35) ^ns^	<0.001
DD ^(P)^	1.30 (1.06, 1.53)	2.26 (1.78, 2.74) ^^P^	1.33 (0.97, 1.69) ^ns^	<0.001

^P^ peak value during treatment or ^N^ nadir value during treatment. ^a^ Estimates and significance of difference between measurements based on GLMM procedure with specification of repeat measures with unstructured covariance structure (linear regression was used for all outcomes except ALC, creatine, and ALT which were highly skewed and required use of gamma regression). Extreme outliers were excluded from the above assessments. ^b^ *p*-value indicates the significance of difference in average values across measurement points. ^^^ *p* < 0.05, comparing average value at each follow-up point to baseline after adjustment for pairwise comparisons using least significant difference. ns = non-significant difference from average value at baseline (*p* > 0.05).

**Table 3:** Adjusted multivariate model a: average values at baseline, min or max during treatment, and at end of treatment ^a^.

	Baseline Mean (95% CI)	Peak ^P^ or Nadir ^N^ Mean (95% CI)	End of Tx Mean (95% CI)	*p*-Value ^b^
WBC (K/UL) ^(N)^	8.0 (7.2, 8.8)	5.2 (4.9, 5.5) ^^N^	7.3 (6.8, 7.8) ^ns^	<0.001
ANC (count/UL) ^(N)^	5215 (4775, 5655)	2587 (2338, 2835) ^^N^	3943 (3544, 4342) ^^^	<0.001
ALC (count/UL) ^(N)^	1868 (1583, 2203)	1348 (1210, 1501) ^^N^	2626 (2413, 2858) ^^^	<0.001
Platelets (K/UL) ^(N)^	235 (222, 249)	204 (191, 216) ^^N^	271 (255, 287) ^^^	<0.001
Creatine (mg/dL) ^(P)^	0.37 (0.34, 0.40)	0.40 (0.38, 0.44) ^^P^	0.34 (0.32, 0.37) ^^^	<0.001
AST (units/L) ^(P)^	57.0 (52.7, 61.3)	78.5 (72.6, 84.3) ^^P^	55.1 (51.0, 59.2) ^ns^	<0.001
ALT (units/L) ^(P)^	41.5 (37.2, 46.2)	57.1 (50.9, 64.1) ^^P^	44.9 (40.2, 50.2) ^ns^	<0.001
Bilirubin ^(P)^	0.56 (0.50, 0.62)	0.67 (0.61, 0.74) ^^P^	0.44 (0.40, 0.48) ^^^	<0.001
INR ^(P)^	1.11 (1.09, 1.13)	1.30 (1.12, 1.43) ^^P^	1.22 (1.09, 1.35) ^ns^	<0.001
DD ^(P)^	1.30 (1.06, 1.54)	2.26 (1.78, 2.74) ^^P^	1.33 (0.97, 1.69) ^ns^	<0.001

^P^ Peak value during treatment or ^N^ nadir value during treatment. ^a^ Estimates and significance of difference between measurements based on GLMM procedure with specification of repeat measures with unstructured covariance structure after adjustment sex (M, F), ethnicity (Hispanic, Non-Hispanic), age (<12, >=12), and having underlying conditions (Y/N to any of the following): [neuro/neuromuscular, immune deficient, oncological, obesity, CLD/asthma, or Other], and days of planned Tx (<=3 vs. >3) (linear regression was used for all outcomes except ALC, creatine, and ALT which were highly skewed and required use of gamma regression). Extreme outliers were excluded from the above assessments. ^b^ *p*-value indicates the significance of difference in average values across measurement points. ^ *p* < 0.05, comparing average value at each follow-up point to baseline after adjustment for pairwise comparisons using least significant difference. ns = non-significant difference from average value at baseline (*p* ≥ 0.05).

**Table 4:** Unadjusted multivariate model: average AST and ALT values at baseline, peak during treatment, and at end of treatment among sub-population of patients whose peak values did not occur at baseline ^a^.

	Baseline Mean (95% CI)	Peak ^P^ Mean (95% CI)	End of Treatment Mean (95% CI)	*p*-Value ^b^
AST (units/L), (n = 191)	49.5 (46.2, 52.8)	84.7 (77.4, 92.1) ^^^	63.5 (57.5, 69.5) ^^^	<0.001
ALT (units/L), (n = 189)	39.3 (34.6, 44.6)	67.1 (58.1, 77.6) ^^^	57.3 (49.8, 65.9) ^^^	<0.001

^P^ Peak value during treatment. ^a^ Estimates and significance of difference between measurements based on GLMM procedure with specification of repeat measures with unstructured covariance structure (linear regression was used for AST and gamma regression for ALT which was highly skewed). Extreme outliers were excluded from the above assessments. ^b^ *p*-value indicates the significance of difference in average values across measurement points. ^ *p* < 0.05, comparing average value at each follow-up point to baseline after adjustment for pairwise comparisons using least significant difference.

## Data Availability

The raw data supporting the conclusions of this article will be made available by the authors on request.

## Abbreviations

The following abbreviations are used in this manuscript:

COVID-19	Coronavirus Disease 2019
SARS-CoV-2	Severe acute respiratory syndrome coronavirus 2
FDA	U.S. Food and Drug Administration
CBC	Complete blood cell count
ANC	Absolute neutrophil count
AST	Aspartate aminotransferase
ALT	Alanine aminotransferase
PT	Prothrombin time
EOT	End of therapy
ULN	Upper limit of normal
GLMM	Generalized linear mixed-effects model
INR	Prothrombin time

## References

[1] Dong Y., Mo X., Hu Y., Qi X., Jiang F., Jiang Z., Tong S. (2020). Epidemiology of COVID-19 Among Children in China. Pediatrics.

[2] DeBiasi R.L., Song X., Delaney M., Bell M., Smith K., Pershad J., Ansusinha E., Hahn A., Hamdy R., Harik N. (2020). Severe Coronavirus Disease-2019 in Children and Young Adults in the Washington, DC, Metropolitan Region. J. Pediatr..

[3] Liguoro I., Pilotto C., Bonanni M., Ferrari M.E., Pusiol A., Nocerino A., Vidal E., Cogo P. (2020). SARS-COV-2 infection in children and newborns: A systematic review. Eur. J. Pediatr..

[4] Lu X., Zhang L., Du H., Zhang J., Li Y.Y., Qu J., Zhang W., Wang Y., Bao S., Li Y. (2020). SARS-CoV-2 Infection in Children. N. Engl. J. Med..

[5] Kim L., Whitaker M., O’Halloran A., Kambhampati A., Chai S.J., Reingold A., Armistead I., Kawasaki B., Meek J., Yousey-Hindes K. (2020). Hospitalization Rates and Characteristics of Children Aged <18 Years Hospitalized with Laboratory-Confirmed COVID-19—COVID-NET, 14 States, March 1–July 25, 2020. MMWR Morb. Mortal. Wkly. Rep..

[6] Beigel J.H., Tomashek K.M., Dodd L.E., Mehta A.K., Zingman B.S., Kalil A.C., Hohmann E., Chu H.Y., Luetkemeyer A., Kline S. (2020). Remdesivir for the Treatment of COVID-19—Final Report. N. Engl. J. Med..

[7] Goldman J.D., Lye D.C.B., Hui D.S., Marks K.M., Bruno R., Montejano R., Spinner C.D., Galli M., Ahn M.Y., Nahass R.G. (2020). Remdesivir for 5 or 10 Days in Patients with Severe COVID-19. N. Engl. J. Med..

[8] Zampino R., Mele F., Florio L.L., Bertolino L., Andini R., Galdo M., De Rosa R., Corcione A., Durante-Mangoni E. (2020). Liver injury in remdesivir-treated COVID-19 patients. Hepatol. Int..

[9] Pettit N.N., Pisano J., Nguyen C.T., Lew A.K., Hazra A., Sherer R., Mullane K.M. (2021). Remdesivir Use in the Setting of Severe Renal Impairment: A Theoretical Concern or Real Risk?. Clin. Infect. Dis..

[10] Chouchana L., Preta L.H., Tisseyre M., Terrier B., Treluyer J.M., Montastruc F. (2021). Kidney disorders as serious adverse drug reactions of remdesivir in coronavirus disease 2019: A retrospective case-noncase study. Kidney Int..

[11] Gerard A.O., Laurain A., Fresse A., Parassol N., Muzzone M., Rocher F., Esnault V.L.M., Drici M.D. (2021). Remdesivir and Acute Renal Failure: A Potential Safety Signal from Disproportionality Analysis of the WHO Safety Database. Clin. Pharmacol. Ther..

[12] Chow E.J., Maust B., Kazmier K.M., Stokes C. (2021). Sinus Bradycardia in a Pediatric Patient Treated with Remdesivir for Acute Coronavirus Disease 2019: A Case Report and a Review of the Literature. J. Pediatr. Infect. Dis. Soc..

[13] Gottlieb R.L., Vaca C.E., Paredes R., Mera J., Webb B.J., Perez G., Oguchi G., Ryan P., Nielsen B.U., Brown M. (2022). Early Remdesivir to Prevent Progression to Severe COVID-19 in Outpatients. N. Engl. J. Med..

[14] Samuel A.M., Hacker L.L., Zebracki J., Bogenschutz M.C., Schulz L., Strayer J., Vanderloo J.P., Cengiz P., Henderson S. (2023). Remdesivir Use in Pediatric Patients for SARS-CoV-2 Treatment: Single Academic Center Study. Pediatr. Infect. Dis. J..

[15] Khalil A., Mohamed A., Hassan M., Magboul S., Ali H., Elmasoudi A.S., Ellithy K., Qusad M., Alhothi A., Al Maslamani E. (2023). Efficacy and Safety of Remdesivir in Hospitalized Pediatric COVID-19: A Retrospective Case-Controlled Study. Ther. Clin. Risk Manag..

[16] Ahmed A., Munoz F.M., Muller W.J., Agwu A., Kimberlin D.W., Galli L., Deville J.G., Sue P.K., Mendez-Echevarria A., Humeniuk R. (2024). Remdesivir for COVID-19 in Hospitalized Children: A Phase 2/3 Study. Pediatrics.

[17] Benfield T., Bodilsen J., Brieghel C., Harboe Z.B., Helleberg M., Holm C., Israelsen S.B., Jensen J., Jensen T.O., Johansen I.S. (2021). Improved Survival Among Hospitalized Patients with Coronavirus Disease 2019 (COVID-19) Treated with Remdesivir and Dexamethasone. A Nationwide Population-Based Cohort Study. Clin. Infect. Dis..

[18] Kautsch K., Wisniowska J., Friedman-Gruszczynska J., Buda P. (2024). Evaluation of the safety profile and therapeutic efficacy of remdesivir in children with SARS-CoV-2 infection—A single-center, retrospective, cohort study. Eur. J. Pediatr..

[19] Player B., Huppler A.R., Pan A.Y., Liegl M., Havens P.L., Ray K., Mitchell M., Graff K. (2024). Safety of remdesivir in the treatment of acute SARS-CoV-2 infection in pediatric patients. BMC Infect. Dis..

[20] Romani L., Roversi M., Bernardi S., Venturini E., Garazzino S., Dona D., Krzysztofiak A., Montagnani C., Funiciello E., Calo Carducci F.I. (2024). Use of Remdesivir in children with COVID-19: Report of an Italian multicenter study. Ital. J. Pediatr..

[21] Morens D.M., Taubenberger J.K., Fauci A.S. (2021). A Centenary Tale of Two Pandemics: The 1918 Influenza Pandemic and COVID-19, Part II. Am. J. Public Health.

